# The clinical and dermatoscopic features of penile pigmentation in men with genital lichen sclerosus

**DOI:** 10.1002/ski2.435

**Published:** 2024-08-11

**Authors:** Mariel L. James, Georgios Kravvas, Aimilios Lallas, Chris B. Bunker

**Affiliations:** ^1^ Department of Dermatology University College Hospitals NHS Foundation Trust London UK; ^2^ First Department of Dermatology School of Medicine Faculty of Health Sciences Aristotle University Thessaloniki Greece

## Abstract

**Background:**

Benign male genital pigmentation is a confusing field with poorly defined terminology. This entity is frequently encountered in our male genital lichen sclerosus (MGLSc) cohort and suggests an association with prior inflammation, however there is a limited literature on the topic.

**Objectives:**

This paper describes the attributes of 21 patients with MGLSc and features of benign genital pigmentation, reviews the existing literature on benign male genital pigmentation and makes recommendations for better practice.

**Methods:**

We prospectively identified 21 patients with MGLSc and clinical diagnoses of benign penile pigmentation who attended specialist male genital dermatoses clinics. Relevant findings were abstracted from clinical notes, outpatient letters, medical photographs, dermatoscopic images and histological reports.

**Results:**

The clinical features of this cohort are discussed and the dermatoscopic images analysed. 15 of 21 patients were followed up for over 2 years and all of these had stable appearance of pigmentation. 87% reported pigmentation to have emerged after the onset of MGLSc symptoms, with latency ranging from one to over 25 years. The terms lentiginosis, melanosis, and post‐inflammatory hyperpigmentation are discussed in context of the existing literature.

**Conclusions:**

We propose that genital lentiginosis and melanosis are clinically indistinguishable macroscopically and are on a clinical and histopathological spectrum. Although there is a compelling narrative that genital melanosis is most often truly benign, there is also emerging evidence to suggest an increased risk of penile melanoma in patients with MGLSc. Furthermore, pigmented lesions in MGLSc can portray concerning morphological features even when benign. A low threshold for biopsy and follow‐up is thus warranted.

## Graphical abstract

This study describes the clinical and dermatoscopic features of 21 patients with benign genital pigmentation (BGM) in the context of male genital lichen sclerosus (MGLSc). BGM is a confusing field of study with poorly defined terminology. We discuss the nomenclature, histopathological features of BGM, its relationship with MGLSc and melanoma and confer our expertise regarding monitoring and performing biopsies.

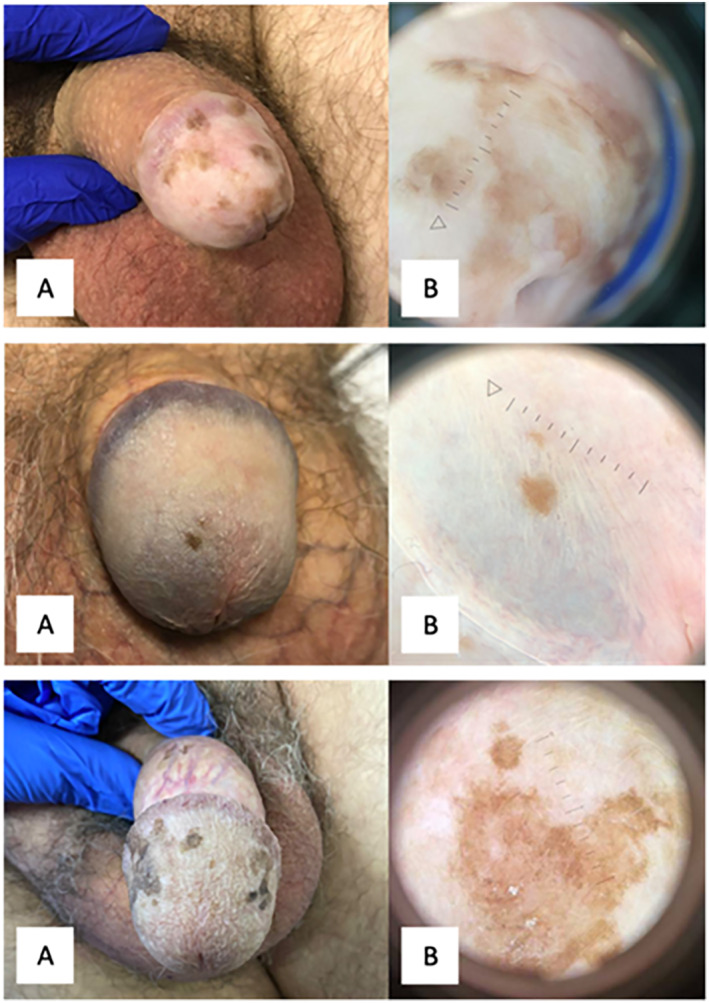

**What's already known about this topic?**

- Benign male genital pigmentation is a confusing field of study with poorly defined terminology. Although terms such as lentiginosis, melanosis, melanoderma and post‐inflammatory hyperpigmentation have utility, their precise aetiopathogenesis, clinicopathological definition and usage in the literature appears inconsistent and not always justifiable. An association with male genital lichen sclerosus (MGLSc) has not yet been established and there is limited literature available on genital pigmentation in the context of this disease.

**What does this study add?**

- This study describes benign genital pigmentation in a series of 21 patients with MGLSc and suggests an association with prior inflammation. We review the available literature and propose that genital lentiginosis and melanosis are overlapping terms that describe a clinical and histopathological spectrum. Although there is a compelling narrative that genital melanosis is most often truly benign, there is also emerging evidence to suggest an increased risk of penile melanoma in patients with MGLSc; thus a high index of suspicion is warranted in this cohort.

## 1 INTRODUCTION

Genital pigmentation is a confusing field of study with poorly defined terminology and can pose a clinical challenge, especially in the context of male genital lichen sclerosus (MGLSc). The challenge has become more important given our recent work showing the high incidence of lichen sclerosus in penile melanoma. Although terms such as lentiginosis, melanosis, melanoderma and post‐inflammatory hyperpigmentation (PIH) have utility, their precise aetiopathogenesis, clinicopathological definition and usage in the literature appears inconsistent and are not always justifiable. This paper describes the clinical and dermatoscopic findings in 21 patients with penile hyperpigmentation, discusses the literature, makes recommendations for better practice and proposes a clinical approach to clarifying the diagnosis in individual cases and the topic in general with enhanced aetiopathological and clinicopathological correlation.

## 2 METHODS

We prospectively identified 21 patients with MGLSc and clinical diagnoses of benign penile pigmentation who attended the specialist male genital dermatoses clinic at University College London Hospitals (17 patients) or who were seen in a specialist private clinic (4 patients) between September and December 2021.

The relevant findings were abstracted from the clinical notes, outpatient letters, medical photographs and histological reports.

The duration of pigmentary changes was calculated based on patients' best estimations of onset or reporting in the medical notes (whichever earliest). The duration of MGLSc symptoms was calculated based on the patient's best estimation, where this could be offered.

## 3 RESULTS

A total of 21 patients with a diagnosis of MGLSc and clinical features of benign penile pigmentation were included. Ages ranged from 33 to 78 years, (median age 49). All patients had clinical and/or histological diagnoses of MGLSc, with active or burnt‐out disease. Eighteen of 21 patients had been circumcised, and one was awaiting circumcision.

The median age at formal diagnosis of MGLSc was 40 years, but symptoms preceded diagnosis in 15 cases (71%). Most patients could not recall precisely when they first noted symptoms of MGLSc or pigmentary changes; therefore, these data represent an estimation. However, eight patients could recall symptoms of MGLSc as far back as childhood or teenagehood. Of the 15 patients, where a quantifiable duration of pigmentation could be estimated, 13 (87%) reported pigmentation to have emerged *after* the onset of MGLSc symptoms, with latency ranging from one to over 25 years. In six of these 13 patients, pigmentation was reported to have emerged after *remission* of MGLSc had been achieved. In these, the lag from remission to pigmentation ranged from 1.5 to 29 years.

We were able to follow up 15 of the 21 patients for over 2 years. The remaining six either had shorter durations of follow‐up (two patients) or did not attend follow‐up (four patients). All followed‐up patients were found to have stable appearances of their pigmentation.

**FIGURE 1:**
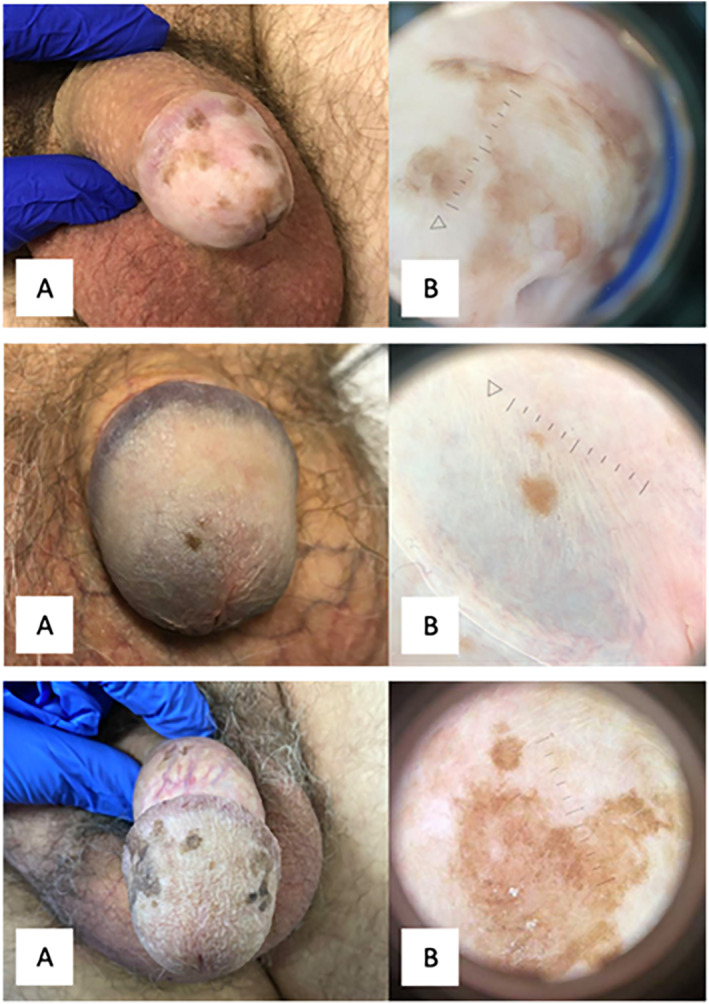
(a) Clinical photographs showing examples of benign genital melanosis. (b) Paired dermatoscopic images.

All pigmented lesions were limited to the glans or inner prepuce, with the vast majority being on the glans. Seven patients reported that their pigmentation emerged gradually and one reported that it had emerged abruptly. The remaining patients could not recall. Most patients reported that pigmentation had been static since first noticed; two reported that it gradually became more prominent when first discussed in 2021; however, at follow‐up review, all 17 patients who re‐attended were noted to have stable pigmentation. Some examples of clinical photographs are shown in Figure 1a.

Dermatoscopic photos were available for 14 patients. One of those patients underwent a diagnostic biopsy due to a background of recurrent undifferentiated (HPV‐driven) penile intraepithelial neoplasia (PeIN) and poorly controlled infection with human immunodeficiency virus. This confirmed the presence of PeIN at the site of pigmentation, despite the lack of clinical suspicion. Subsequent treatment with a combination of imiquimod, podophyllotoxin and liquid nitrogen cryotherapy has led to markedly reduced pigmentation in the area. In this patient, initial dermatoscopy revealed numerous brown dots/globules arranged in a linear fashion. Of the remaining 13 patients, lesions showed dermatoscopic symmetry in seven and asymmetry in six. Brown was the predominant colour in all of them, and no additional colours were observed. The predominant pattern was structureless in six cases, parallel lines in five cases, and circles in two cases. The most frequent local features were structureless areas (8/13) and parallel lines (8/13), while circles and dots/globules were seen in 4 out of 13 and 3 out of 13 cases, respectively. Vascular structures were not observed in any of the lesions. Some examples of dermatoscopic photos are presented in Figure 1b.

A summary of all the findings is given in Table 1.

**TABLE 1:** The clinical features of all the patients included in this study.

Patient number	Age	Age at symptoms onset of MGLSc (years since symptom onset)	Age at formal diagnosis of MGLSc	Age at circumcision (years since circumcision)	Active symptoms of MGLSc	Years since last active symptoms of MGLSc	Years since onset of pigmentary change (estimated)	Pigmentary change before or after the *onset* of MGLSc	Pigmentary change during active MGLSc or after remission	Dermatoscopic images	Unifocal or multifocal	Speed of onset of pigmentary change	Evolution of pigmentary change at initial index visit	Follow up ≥ 2 years from index appointment (and nature of pigmentation at the final follow‐up)	Histology of pigmentary change
1	35	28 (7)	29	29 (6)	Yes	Ongoing	5	After	During	No	Multifocal	Unknown	Static	Yes (stable)	No
2	40	Childhood	40	8 (32)	No	32	3	After	After	Yes	Multifocal	Unknown	Unknown	Yes (stable)	No
3	37	Late teens	30	30 (7)	No	4	2	After	After	No	Multifocal	Gradual	Static	Yes (stable)	No
4	33	Childhood	24	24 (9)	No	9	1	After	After	Yes	Multifocal	Abrupt	Static	Yes (stable)	No
5	53	25 (28)	45	48 (5)	No	5	5	After	After	Yes	Multifocal	Gradual	Gradually becoming darker	Yes (stable)	No
6	58	43 (15)	44	44 (14)	Yes	Ongoing	3	After	During	No	Multifocal	Unknown	Static	Yes (stable)	No
7	55	Unknown	39	39 (16)	Unknown	Unknown	5	After	Unknown	Yes	Multifocal	Unknown	Unknown	Yes (stable)	No
8	49	41 (8)	40	45 (8)	Yes	Ongoing	7	After	During	Yes	Multifocal	Gradual	Static	Yes (stable)	uPeIN
9	60	Unknown	55	55 (5)	No	Unknown	2	After	Unknown	Yes	Multifocal	Unknown	Unknown	Yes (stable)	No
10	43	Unknown	Unknown	30 (13)	Yes	Ongoing	Unclear	Unknown	Unknown	Yes	Multifocal	Unknown	Unknown	No (18 months follow up, stable)	No
11	78	67 (11)	67	67 (11)	No	11	2	After	After	Yes	Unifocal	Gradual	Static	Yes (stable)	No
12	50	Childhood	49	n/a	Yes	Ongoing	Unclear	Unknown	Unknown	Yes	Unifocal	Unknown	Static	Yes (stable)	No
13	39	Childhood	38	n/a	Yes	Ongoing	Unclear	Unknown	Unknown	Yes	Multifocal	Unknown	Static	No (8 months of follow‐up, stable)	No
14	62	35 (27)	58	59 (3)	No	3	4	After	During	Yes	Multifocal	Unknown	Static	Yes (stable)	No
15	75	Denied symptoms	75	Infancy	No	n/a	≥10	Unknown	Unknown	Yes	Multifocal	Gradual	Static	Yes (stable)	No
16	57	52 (5)	55	55 (2)	No	2	<1	After	After	Yes	Multifocal	Unknown	Static	Yes (stable)	No
17	35	Childhood	30	30 (5)	Yes	Ongoing	Unclear	Unknown	Unknown	Yes	Unifocal	Unknown	Static	Yes (stable)	No
18	51	49 (2)	50	50 (1)	No	1	Unclear	Unknown	Unknown	No	Multifocal	Unknown	Static	No	No
19	38	14 (24)	37	n/a	Yes	Ongoing	5	After	During	No	Multifocal	Gradual	Static	No	No
20	34	Childhood	33	10 (24)	No	24	24	Unknown	Unknown	No	Unifocal	Unknown	Static	No	No
21	42	33 (9)	36	42 (0)	Unknown	Unknown	Unclear	Unknown	Unknown	No	Multifocal	Gradual	Gradually becoming darker and expanding in area	No	No

Abbreviations: MGLSc, Male genital lichen sclerosus; uPeIN, undifferentiated penile intraepithelial neoplasia.

## 4 DISCUSSION

Genital pigmentary disorders constitute a confusing field in the medical literature, with a lack of clear distinction between different entities. Particular confusion lies between the use of the terms PIH, melanosis and lentiginosis.

### 4.1 Post‐inflammatory hyperpigmentation

PIH is the development of localised hyperpigmentation as a consequence of prior inflammation. Epidermal PIH is characterised by an increase in melanin synthesis and its transfer to the surrounding keratinocytes.^1^ It usually resolves spontaneously, but this can take months or even years.^1^ Dermal PIH occurs where there is damage to basal keratinocytes and may be referred to as dermal melanosis or pigment incontinence.^1^ Dermal PIH has a more bluish‐grey appearance and may be slower to resolve or remain permanently.^1^ Dermatoscopy of PIH is not well described but has been reported to demonstrate light to dark brown structureless areas.^2^

### 4.2 Melanosis and lentiginosis

Benign genital pigmentation has been variously labelled with the terms lentiginosis and melanosis. However, some authors have suggested that these are separate entities.^3^, ^4^

Penile *melanosis* is frequently described as the cause of pigmented macules; it is often large, with well‐defined edges, occasionally irregular, often multifocal and with variegated pigmentary patterns.^4^ A linear variant has also been reported.^5^ The term ‘benign melanotic macules’ was suggested by Lenane for smaller, discrete areas of melanosis.^6^ It is not uncommon for melanosis to have features that raise concerns about atypical melanocytic proliferations or melanoma warranting biopsy.^7^ The aetiopathogenesis is not well understood, although some authors characterise melanosis as pigmentation with no overt evidence of preceding inflammation.^8^

Accounts of penile *lentiginosis* describe pigmented macules that can occur idiopathically or in conjunction with a ‘lentiginosis’ syndrome, such as Laugier–Hunziker syndrome, Peutz–Jeghers syndrome, LAMB syndrome (lentigines, atrial myxomas, mucocutaneous myxomas, and blue naevi), Noonan syndrome with multiple lentigines, and Ruvalcaba‐Mhyre‐Smith.^3^ However, classical textbooks describe Laugier–Hunziker syndrome as a cause of *melanosis.* ^3^, ^8^ Others, such as Revuz et al., describe lentiginosis together with melanosis under the umbrella of ‘essential melanotic hyperpigmentation of the mucosa’.^9^

In vulval dermatology, melanosis and lentiginosis are accepted to be synonymous but this same consensus has not yet been achieved in penile dermatology, although the terms are frequently used interchangeably.^10^ To further support the propinquity of the two terms, papers that focus on lentiginosis have often been cited in newer publications on *melanosis*, indicating that contemporary authors interpret the terms as equivalent.

Similarly, from a macroscopic perspective, lentigines and melanosis are universally described.^11^, ^12^ Dermatoscopic descriptions of penile melanosis feature parallel brown lines, ring‐like patterns, reticular‐like patterns or structureless patterns,^13^ and similar appearances are described for vulval melanosis/lentiginosis.^10^ Confusingly, melanosis and lentiginosis might also be considered a form of PIH; indeed, a popular Internet resource (Dermnetnz.org) describes PIH as ‘acquired melanosis’.^14^

There is no consensual histological definition of the terms ‘lentiginosis’ and ‘melanosis’. Barnhill et al. suggested that ‘lentiginosis’ features elongation of rete ridges with basal layer hyperpigmentation, epithelial hyperplasia and basal melanocyte hyperplasia without atypia.^12^ Breathnach et al. proposed that depigmentation is an essential characteristic of lentiginosis due to lymphocytic destruction of melanocytes.^11^ Revuz et al. proposed that melanosis lacks melanocyte hyperplasia.^9^ However, both McKee's^8^ and Rook's^4^ textbooks suggest that there *may* be lentiginous melanocytic hyperplasia in melanosis. This may make isolated benign melanotic macules (melanosis) difficult to distinguish from what may be thought of as ‘lentigines’ or even early junctional naevi. Barnhill et al. suggested in 1992 that ‘lentigo’ was an appropriate term for lesions with lentiginous melanocytic hyperplasia, and that ‘melanosis’ had (in 1992) been most typically applied to vulval lesions with basal layer hyperpigmentation with *or* without melanocytic hyperplasia.^12^ In their histologically assessed case series, most pigmented vulval/penile lesions displayed slight basal melanocytic hyperplasia, and thus they settled on the term ‘lentiginosis’, but suggested that melanosis could be an acceptable term for lesions without melanocytic hyperplasia.^12^

Given the lack of consensus, and the macroscopic and histopathological overlap, the distinction to us seems arbitrary and unhelpful. We therefore propose that it is reasonable to consider melanosis and lentiginosis in continuity. Furthermore, the compelling conclusion is that, however defined, these lesions are benign.

Based on the above statements, we have elected to refer to these conditions as *benign genital melanosis* (BGM) for the remainder of this discussion.

### 4.3 Benign genital melanosis versus post‐inflammatory hyperpigmentation

BGM may be difficult to distinguish clinically from PIH as both can occur following inflammation and can present with pigmented, dermatoscopically structureless macules. Traditionally, BGM is thought to develop slowly, whereas PIH may be seen more acutely following inflammation. We observed a lag of up to 29 years since the last active symptomatic phase of MGLSc prior to the development of pigmentation. Although in one case macules were felt to have appeared quite suddenly, the remainder of the patients described a gradual onset. BGM may be more likely to present with well‐defined macules such as in most of our cohort, whereas PIH may present with more diffuse or widespread change. PIH may be more common in darker‐skinned individuals, but our cohort was predominantly fair‐skinned. This reflects the publication by Barnhill et al. in which all patients were Caucasian.^12^ Another distinguishing feature is that BGM macules seem to persist, often with no evidence of fading as may be expected with PIH. Our experience is that BGM does not resolve.

Given our lack of histopathological data, it is feasible that some of these lesions represent PIH. It is, however, also possible that biopsies would not differentiate between the two conditions, as there is a histological crossover. Overall, we have felt that our cohort has demonstrated BGM rather than PIH, but this has to be speculative and a paucity of specifically directed histology contributes to the lack of certainty.

### 4.4 Genital pigmentation and MGLSc

Cases of BGM have been documented in association with dithranol, psoralen ultraviolet light, and in diabetes.^12^, ^15^ However, there is limited literature on the possible association of BGM with prior inflammation, such as from MGLSc.

There is but one case report specifically commenting on ‘melanosis’ associated with MGLSc; this describes a 74‐year‐old with asymptomatic hyperpigmentation on the glans where histopathological examination revealed ‘melanosis/lentiginosis’, with no cytological atypia and no melanocytic nests, alongside features of lichen sclerosus.^16^ El Shabrawi‐Caelen et al. published on ‘lentigines’ associated with MGLSc and found that histological features may simulate the appearance of a regressed melanoma and could create diagnostic confusion.^17^ Sollena et al. published a report on an incidental asymptomatic pigmented lesion on the glans, which was dermatoscopically suspicious for melanoma with a composite pattern of globules and streaks with ill‐defined borders associated with hypopigmented areas. Histology showed features of LSc and melanophages in the papillary dermis, and a diagnosis of PIH was made.^18^

Therefore, neither lentiginosis, melanosis, nor PIH feature prominently in the literature in association with MGLSc. However, the paucity of publications does not reflect the frequency of BGM that we encounter in our designated penile dermatology clinic.

Cullen's 1962 study reported that 14.2% of 10 000 men had penile ‘naevi’^19^; however, this did not incorporate histopathological examination nor comment on prior inflammation. Therefore, the frequency of true melanocytic naevi versus BGM is unclear, as is any relationship with MGLSc. In the case series by Barnhill et al., 30% of pigmented genital macules were deemed related to prior injury or irritation.^12^ Our case series is not powered to comment on incidence or prevalence, but we have been struck by the frequency of BGM in our overall cohort of patients with MGLSc, with the context that our department almost never sees cases of BGM outside of the MGLSc cohort. Of course, this observation is modulated by the fact that patients without an underlying inflammatory disorder may not present to services.

The mechanisms by which MGLSc may cause BGM distinct from PIH are not well described or discussed. Proposed mechanisms for post‐inflammatory pigmentary changes include melanocyte damage or loss resulting in melanin spillage and melanocyte stimulation by cytokines or hormones.^3^ Our working theory is that melanocytic perturbation and distress from MGLSc may result in pigmentary dysfunction even years after the inflammation has ceased. Further research is clearly warranted into the effects of MGLSc on melanocytes, especially given the emerging relationship with atypical melanocytic proliferations and melanoma (vide infra).^20^, ^21^

### 4.5 Benign genital melanosis and melanoma

There are limited published data on the progression of BGM, but the little literature that exist suggests that most lesions do not progress or grow.^6^, ^9^, ^12^, ^22^ Haugh et al. performed a retrospective review and histological analysis of 41 men and women with genital melanosis with an average follow‐up period of 30.5 months. They suggested that genital melanosis is unlikely to present an imminent risk for genital melanoma and recommended close clinical follow‐up without the need for routine biopsies.^22^ Reassuringly, the patients described in our case series lacked red flag symptoms, for example, rapid growth, underlining the essential benign pathogenesis and prognosis of their pigmentary lesions.

**FIGURE 2:**
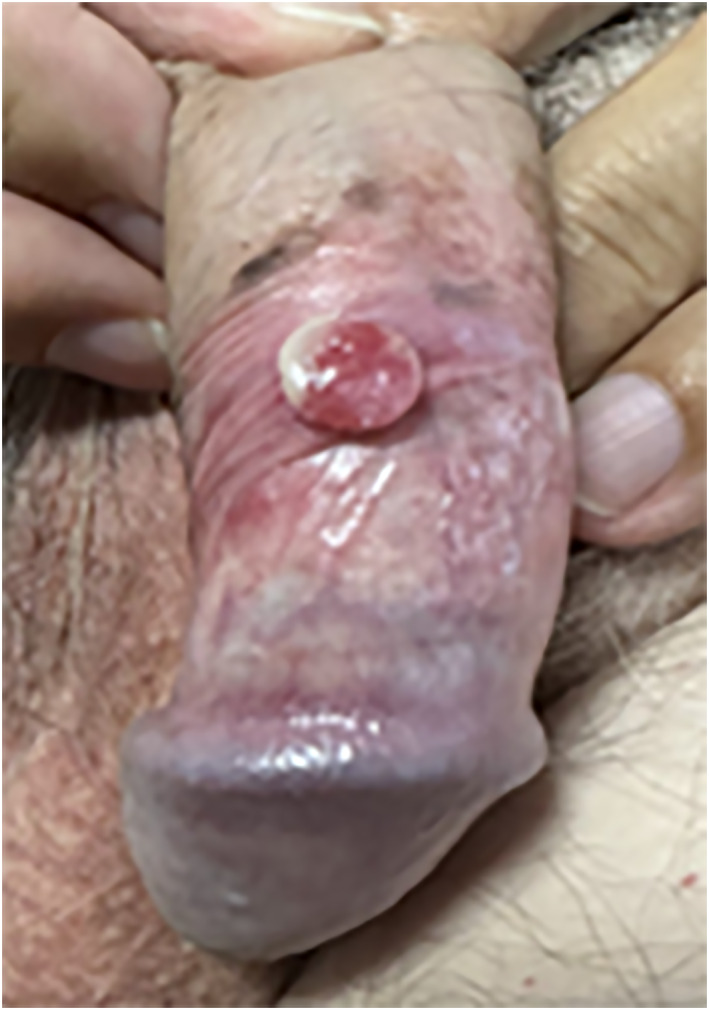
A case of penile melanoma with a background of extensive melanoma in situ and male genital lichen sclerosus.

Beyond PIH and BGM, the possibility of true melanocytic proliferations of the penis must not be overlooked. Benign penile naevi in the context of MGLSc can be mistaken clinically for melanoma.^23^ Melanocytic naevi associated with MGLSc can also mimic melanoma *histologically*; those emerging on a background of vulval LSc have been shown to exhibit an activated phenotype with melanocytes containing abundant melanin, proliferating contiguously with fibrotic collagen and featuring higher Ki‐67 labelling index than control naevi.^24^ In male genitalia, benign melanocytic naevi associated with MGLSc have been found to feature confluent nests varying in size and shape, with pagetoid spread of nests and single melanocytes.^17^ Masquerades aside, a possible increased risk of melanoma is an important consideration in this cohort.^20^, ^21^, ^25^, ^26^ Recent observations from our centre showed that 82% of cases of penile melanoma over an 11‐year period showed background histological features of MGLSc. Since then, we have seen a further case of penile melanoma (Figure 2) with background extensive melanoma in situ and MGLSc. Although causation has not been established, these findings reinforce the importance of surveillance and a higher index of suspicion. We advocate that dermatoscopy should be undertaken for all pigmented genital lesions with active or past MGLSc. Clinicians and patients need to be aware of the importance of detecting and monitoring pigmentary changes. We propose that photographic monitoring alongside self‐monitoring advice is a condign management stratagem for most patients.

### 4.6 BGM and diagnostic biopsies

Where there is any reason for suspicion, biopsy is essential, such as for Case 8 where a diagnosis of PeIN was made. This finding underlines a well‐known morphological propensity of intraepithelial neoplasia and skin cancer to present with pigmentation. Other than PeIN, genital nonmelanoma skin cancers, such as pigmented basal cell carcinoma and squamous cell carcinoma, have also been described in the literature. Although these are rarely encountered in clinical practice, they should be considered in the differential diagnosis of atypical, pigmented genital lesions.^27^

Whilst we acknowledge that the threshold for biopsy should be low, avoidance of unnecessary biopsies has been a key tenet of our approach to patients with reassuring macroscopic and dermatoscopic presentations. Patients with MGLSc may have disease‐related anatomical disruption and may be subjected to diagnostic and therapeutic surgical procedures. It is therefore important for their psychological and sexual well‐being to limit unnecessary interference. All this notwithstanding, we do acknowledge that our threshold for biopsy has changed, and we wish to communicate this to others. Reassuringly, all 16 patients that we have been able to follow up showed no worsening of their pigmentary changes for a period of over two years.

### 4.7 Treatment of BGM

Treatment options for BGM are limited. Lasers have been found to be successful but are not widely performed.^28^ Following our reassurance, none of our patients actively sought treatment for their pigmentation, and nor did we identify any robust theme of psychosocial impact specifically from the pigmented lesions.

### 4.8 Limitations of this study

This case series has a number of limitations, the first being a lack of histological data which makes it impossible to diagnose definitively the pigmented lesions seen in our cohort. However, given the descriptions of lentiginosis and melanosis obtained from the existing literature, and the long period of follow‐up, we are confident that the diagnosis was highly likely to be BGM in 20 out of 21 cases. The clinical observation that BGM appears more commonly in men with MGLSc cannot be proven given that (a) we do not have epidemiological data (and nor does this exist in the literature) and (b) we do not have data from controls. Our dataset lacks precision, particularly concerning the estimated nature of symptom duration (both for MGLSc and pigmentation). However, our findings suggest that most pigmentary changes appeared after the emergence of MGLSc. Large prospective studies would add support to these theories.

## 5 SUMMARY

In summary, we describe a cohort of 21 patients who manifested penile pigmentary changes in the context of MGLSc. We have reviewed the literature and concluded that the existing nomenclature for benign genital pigmentation is confusing and lacks clear definition. Penile melanosis and lentiginosis appear to be a part of the same continuum and thus we propose that they should be considered the same entity, *viz* BGM. Moreover, we propose that BGM is the clinical diagnosis in 20 of our cases and that there must be a relationship between MGLSc and BGM. However, more work on the clinicopathological correlation and terminology of penile pigmentation is needed. The onset of BGM may display a long latency since any active inflammation from MGLSc, and this points to a possible pathophysiology involving chronic melanocytic memory of damage and distress. Further research is needed into the aetiopathogenesis of pigmentary phenomena in association with MGLSc. There is a compelling narrative that BGM is most often truly benign; thus, for clinically and dermatoscopically benign‐appearing lesions, we recommend reassurance and/or monitoring with serial clinical and dermatoscopic photographs. However, we also acknowledge that pigmented lesions in MGLSc can portray concerning morphological features even when benign. Given the emerging evidence that genital melanoma may be more tightly associated with MGLSc than previously thought, biopsies should always be considered and clinical suspicion heightened.

## CONFLICT OF INTEREST STATEMENT

None to declare.

## AUTHOR CONTRIBUTIONS

**Mariel L. James**: Formal analysis (equal); writing – original draft (lead); writing – review & editing (equal). **Georgios Kravvas**: Formal analysis (equal); supervision (supporting); writing – original draft (supporting); writing – review & editing (equal). **Aimilios Lallas**: Formal analysis (equal); writing – original draft (supporting); writing – review & editing (equal). **Chris B. Bunker**: Formal analysis (equal); supervision (lead); writing – original draft (equal); writing – review & editing (lead).

## ETHICS STATEMENT

The patients in this manuscript have given written informed consent to the publication of their case details.

## PATIENT CONSENT

Written patient consent was obtained for publication.

## Data Availability

Data sharing is not applicable.
